# Type I Interferons Induce T Regulatory 1 Responses and Restrict Humoral Immunity during Experimental Malaria

**DOI:** 10.1371/journal.ppat.1005945

**Published:** 2016-10-12

**Authors:** Ryan A. Zander, Jenna J. Guthmiller, Amy C. Graham, Rosemary L. Pope, Bradly E. Burke, Daniel J.J. Carr, Noah S. Butler

**Affiliations:** 1 Departments of Microbiology and Immunology, University of Oklahoma Health Sciences Center, Oklahoma City, Oklahoma, United States of America; 2 Department of Ophthalmology, University of Oklahoma Health Sciences Center, Oklahoma City, Oklahoma, United States of America; 3 Graduate Program in Biosciences, University of Oklahoma Health Sciences Center, Oklahoma City, Oklahoma, United States of America; McGill University, CANADA

## Abstract

CD4 T cell-dependent antibody responses are essential for limiting *Plasmodium* parasite replication and the severity of malaria; however, the factors that regulate humoral immunity during highly inflammatory, Th1-biased systemic infections are poorly understood. Using genetic and biochemical approaches, we show that *Plasmodium* infection-induced type I interferons limit T follicular helper accumulation and constrain anti-malarial humoral immunity. Mechanistically we show that CD4 T cell-intrinsic type I interferon signaling induces T-bet and Blimp-1 expression, thereby promoting T regulatory 1 responses. We further show that the secreted effector cytokines of T regulatory 1 cells, IL-10 and IFN-γ, collaborate to restrict T follicular helper accumulation, limit parasite-specific antibody responses, and diminish parasite control. This circuit of interferon-mediated Blimp-1 induction is also operational during chronic virus infection and can occur independently of IL-2 signaling. Thus, type I interferon-mediated induction of Blimp-1 and subsequent expansion of T regulatory 1 cells represent generalizable features of systemic, inflammatory Th1-biased viral and parasitic infections that are associated with suppression of humoral immunity.

## Author Summary

Humoral immunity is essential for host resistance to pathogens that trigger highly inflammatory immune responses, including *Plasmodium* parasites, the causative agents of malaria. Long-lived, secreted antibody responses depend on a specialized subset of CD4 T cells called T follicular helper (Tfh) cells. However, anti-*Plasmodium* humoral immunity is often short-lived, non-sterilizing, and immunity rapidly wanes, leaving individuals susceptible to repeated bouts of malaria. Here we explored the relationship between inflammatory type I interferons, the regulation of pathogen-specific CD4 T cell responses, and humoral immunity using models of experimental malaria and systemic virus infection. We identified that type I interferons promote the formation and accumulation of pathogen-specific CD4 T regulatory 1 cells that co-express interferon-gamma and interleukin-10. Moreover, we show that the combined activity of interferon-gamma and interleukin-10 limits the magnitude of infection-induced Tfh responses, the secretion of parasite-specific secreted antibody, and parasite control. Our study provides new insight into the regulation of T regulatory 1 responses and humoral immunity during inflammatory immune reactions against systemic infections.

## Introduction

Malaria, caused by mosquito-borne *Plasmodium* parasites, remains a significant burden on public health that is responsible for over 400,000 deaths annually [1]. Immunological studies in humans and mice have identified parasite-specific antibodies as critical for *Plasmodium* control and parasite clearance [2]. However, an abundance of data show that antibody responses generated against *Plasmodium* parasites are relatively short-lived and dominated by antibodies of low affinity [3–6], which leaves individuals susceptible to repeated infection [2, 7]. Despite these long-standing observations, the infection-induced, host-specific factors that limit the acquisition of long-lived anti-*Plasmodium* antibody responses following single or repeated *Plasmodium* infection remain poorly defined.

T follicular helper (Tfh) cells are essential for the generation of memory B cells and plasma cells that produce high-affinity antibodies, two B cell subsets that comprise long-lived humoral immunity against pathogen reinfection [8, 9]. Tfh cells functionally orchestrate germinal center (GC) B cell reactions through ligand-receptor interactions and cytokine secretion [10]. The importance of Tfh cells in promoting antibody-mediated control of numerous acute and chronic infections is well established [10–12]. However, less is known about *Plasmodium*-specific Tfh cell differentiation, maintenance, and function, despite the critical role of *Plasmodium*-specific secreted antibodies in limiting disease severity and promoting parasite clearance. Indeed, numerically or functionally skewed pathogen-specific Tfh responses represent an emerging hypothesis to explain the defects or delays in the acquisition of antibody-mediated immunity following infection [13].

In support of this hypothesis, a recent survey of Tfh cell responses in *Plasmodium falciparum*-exposed children revealed that a phenotypically and functionally distinct T helper 1 (Th1)-like Tfh cell subset (CXCR3^+^ CXCR5^+^PD-1^+^) is preferentially expanded during human malaria [14]. Strikingly, these Th1-like Tfh cells exhibit a markedly reduced capacity to provide B cell help *in vitro* and the expansion of this subset was further linked to Th1-associated, *Plasmodium* infection-induced inflammation [14]. In agreement with the later observation, we originally reported that excessive type II IFN (IFN-γ-associated inflammation impairs Tfh activity and humoral immunity during experimental malaria [15], a finding recently confirmed by others [16]. Together, these data support that Tfh responses generated during malaria may be suboptimal, and that the inflammatory environment or cytokine milieu induced by *Plasmodium* blood-stage infection can impact the quantity or quality of anti-*Plasmodium* Tfh cell responses with subsequent impacts on humoral immunity.

In addition to Th1-associated inflammation and systemic production of IFN-γ, type I interferons (IFNα/β) are also highly induced during human and experimental blood-stage *Plasmodium* infection [17–22]. Type I IFNs are pleiotropic cytokines with reported variable effects on Tfh development and function. During acute viral infection, type I IFNs suppress the Tfh developmental program [23]. On the other hand, in vitro studies show STAT1-dependent, type I IFN receptor (IFNAR) signaling can promote Tfh cell differentiation [24]. To date, the functional roles of type I IFNs during *Plasmodium* infection have mainly focused on acutely lethal, experimental cerebral malaria (ECM) models. In this context, IFNAR signaling suppressed Th1 development and activity, which led to elevated parasite burdens and exacerbated malaria-induced neurological disease [17, 25]. However, the contribution of type I IFNs in regulating *Plasmodium*-specific Th1 and/or Tfh activity and parasite-specific antibody responses during non-lethal experimental malaria have not been extensively investigated.

Here we used complementary genetic and biochemical approaches to test the hypothesis that IFNAR signaling represents an additional inflammatory signaling cascade that limits the quantity and quality of *Plasmodium*-specific Tfh cell responses and the subsequent generation of protective humoral immunity. Using both chronic viral and parasitic infection models, we uncovered a molecular circuit in which type I IFNs directly induce Blimp-1 expression in pathogen-specific CD4 T cells and promote T regulatory 1 responses. Furthermore, we identified that type I IFN-mediated induction of Tr1- associated cytokines IL-10 and IFN-γ collaborate to limit the generation of protective humoral immunity during experimental malaria.

## Results

### Type I IFNs limit parasite control, germinal center B cell and secreted antibody responses during experimental *Plasmodium* infection

**Fig 1:**
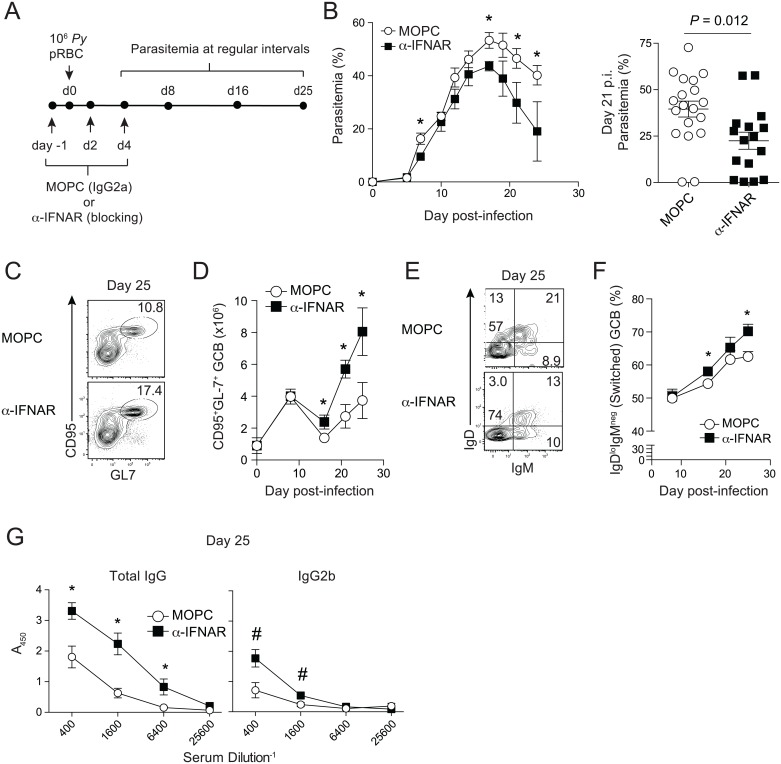
Blockade of IFNAR signaling improves parasite control and enhances humoral immunity during *P*. *yoelli* infection. (**A**) Experimental design. Mice were administered either MOPC (isotype) or α-IFNAR antibodies at the indicated time points and were infected with 10^6^ *P*. *yoelii* infected red blood cells (pRBCs). (**B**) Parasitemia (% of RBCs infected) kinetics (left) and cumulative data from 4 independent experiments displaying parasitemia on day 21 p.i. (right). (**C**) Representative flow plots from MOPC (top) or α-IFNAR-treated mice (bottom) depicting the proportion of CD95^+^GL7^+^CD19^+^ splenic germinal center (GC) B cells on day 25 p.i. (**D**) Summary kinetics displaying the total numbers of GC B cells. (**E,F**) Representative flow plots (E) and summary data (F) showing the proportion of class-switched (IgD^-^IgM^-^) GC B cells (**G**) Summary graphs displaying the relative titers of MSP1_19_-specific total IgG (left) and IgG2b (right) on day 25 p.i. Data (Mean +/- SEM) in (**B,D,F,G**) are pooled from 2–3 independent experiments per time point (3–5 mice/group for each experiment) and were analyzed using Mann-Whitney (non-parametric) tests of statistical significance. Data in (**B-G**) are representative of >3 independent experiments. *P<0.05, ^#^P = 0.05.

To begin to dissect the biological effects of type I IFNs during *Plasmodium* blood-stage infection [17–22], we employed reagents to block IFNAR signaling in a model of non-lethal experimental *P*. *yoelii* malaria. We administered to *P*. *yoelii*-infected mice either an irrelevant rat IgG (clone MOPC) or an extensively characterized monoclonal antibody (mAb, clone MAR-15A3) that blocks signaling from the type I IFN receptor (IFNAR) [26–28] (Fig 1A). Blocking IFNAR signaling through day 4 p.i. resulted in a 25–50% decrease in *P*. *yoelii* parasitema after day 16 p.i. (Fig 1B). By contrast, blocking IFNAR signaling during the second week of infection (day 10, 12, 14 p.i.) had no impact on parasite control (S1A Fig). These data demonstrate that early type I IFN responses impede parasite control during experimental *Plasmodium* blood-stage infection.

Notably, blocking IFNAR signaling through day 4 p.i. largely did not impact parasite replication until the second week of infection (Fig 1B), which is consistent with our hypothesis that type I IFNs negatively impact the development of humoral immunity. Thus, we next examined the magnitude and kinetics of *Plasmodium* infection-induced GC B cell responses in MOPC- and α-IFNAR-treated mice. By the third week of experimental malaria, α-IFNAR treatment resulted in 2 to 4-fold increases in the number of *P*. *yoelii* infection-induced (S1B Fig) splenic GL7^+^CD95^+^ CD19^+^ GC B cells (Fig 1C and 1D). IFNAR signaling blockade was also associated with enhanced class-switching in GC B cells (Fig 1E and 1F). The quantitative and qualitative changes in responding B cells in α-IFNAR-treated mice were additionally linked to 2-fold higher serum titers of T cell-dependent, merozoite surface protein 1 (MSP1_19_)-specific antibody (Fig 1G). Collectively, these results show that early type I IFN signaling negatively regulates parasite control and the quantity and quality of humoral immunity during experimental *Plasmodium* infection.

### Type I IFNs limit CD4+ T follicular helper cell accumulation and promote T regulatory 1 responses during experimental malaria

**Fig 2:**
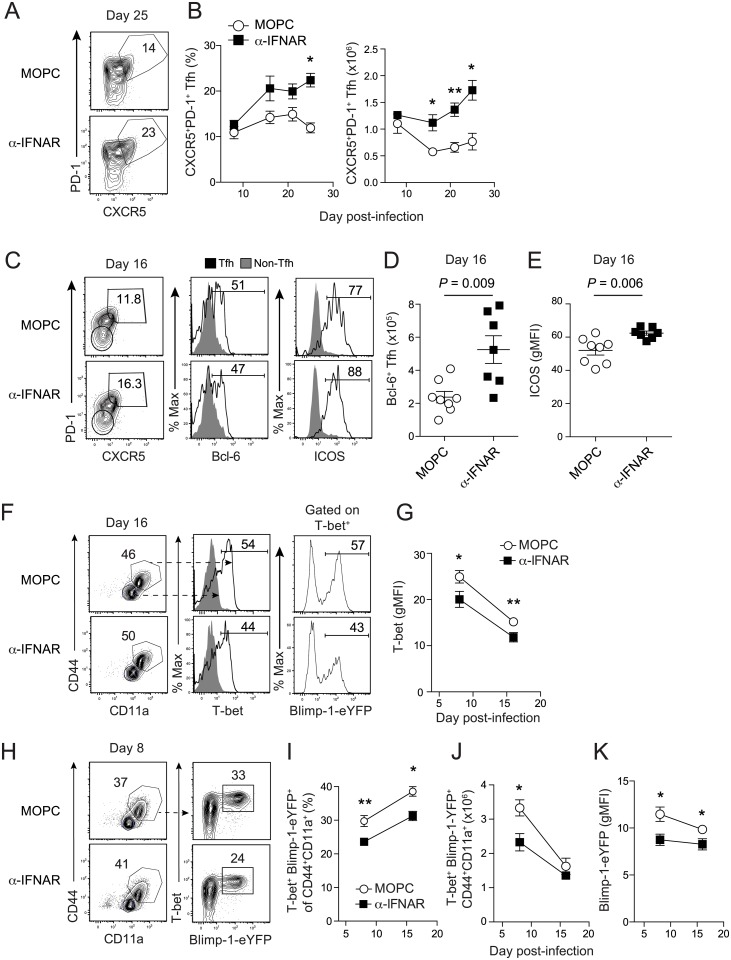
Blockade of IFNAR signaling enhances Tfh cell accumulation and limits T regulatory 1 responses during experimental malaria. (**A-K**) Wild type (WT) mice were administered either MOPC or α-IFNAR antibodies and were infected with 10^6^ pRBCs. (**A**) Representative flow plots from day 25 p.i. depicting the percentage of CXCR5^+^PD-1^+^ Tfh cells among splenic CD44^+^ CD4 T cells from MOPC and α-IFNAR-treated mice. (**B**) Summary kinetics displaying the proportion (left) and total number (right) of Tfh cells from MOPC and α-IFNAR-treated mice. Representative flow plots (**C**) and summary data depicting total numbers of CXCR5^+^PD-1^+^Bcl-6^+^ Tfh (**D**) and ICOS (**E**) expression (geometric MFI) on Tfh cells from MOPC and α-IFNAR-treated mice on day 16 p.i. (**F-K**) Blimp-1-eYFP reporter mice were administered either MOPC or α-IFNAR antibodies and were infected with 10^6^ pRBCs. (**F**) Representative dot plots (left) depicting the proportion of splenic CD44^+^CD11a^hi^ CD4 T cells in MOPC and α-IFNAR-treated mice expressing either T-bet (middle) or Blimp-1-eYFP in T-bet^+^ CD4 T cells (right). (**G**) Summary kinetics depicting the relative expression of T-bet (gMFI) among *Plasmodium* infection-induced CD44^+^CD11a^+^ CD4 T cells. (**H**) Representative flow plots from MOPC and α-IFNAR-treated mice on day 8 p.i showing the proportion of splenic CD44^+^CD11a^hi^ CD4 T cells simultaneously expressing both T-bet and Blimp-1-eYFP. (**I-J**) Summary kinetics depicting the frequency (I) and total number (J) of T-bet^+^Blimp1-eYFP^+^ splenic CD44^+^CD11a^hi^ CD4 T cells in MOPC and α-IFNAR-treated mice. (**K**) Summary kinetics of Blimp-1-eYFP expression (gMFI) in splenic CD44^+^CD11a^hi^ CD4 T cells in MOPC and α-IFNAR-treated mice. Data (Mean +/- SEM) in (**A,D,G,J-K**) are pooled from 2–3 independent experiments per time point (3–5 mice/group for each time point) and were analyzed using Mann-Whitney (non-parametric) tests of statistical significance. *N*.*S*. = not statistically significant. *P<0.05, **P<0.01.

IFNAR signaling is reported to regulate the proliferation, survival and differentiation of multiple effector subsets [29], including T follicular helper (Tfh) cell responses in vitro [24] and *in vivo* following immunization with model antigens [30]. Type I IFNs have also been shown to indirectly regulate Th1 CD4 T cell activity during acutely lethal experimental cerebral malaria and acute virus infection [25, 27, 28]. Thus, we next explored whether IFNAR signaling blockade differentially impacts the differentiation of Tfh or Th1 cells during prolonged *P*. *yoelii* blood stage malaria. Of note, the total *Plasmodium* infection-induced effector CD4 T cell compartment can be distinguished from irrelevant (naïve) CD4 T cells via published surrogate marker approaches that monitor conformational changes in CD11a and upregulation of CD44 on CD4 T cells following infection or vaccination [15, 31–36]. Consistent with enhanced humoral immunity, we found α-IFNAR treatment resulted in 2 to 3-fold expansions in the frequency and total number of *Plasmodium*-infection-induced (S1C Fig) splenic PD-1^+^CXCR5^+^Bcl-6^+^ Tfh cells (Fig 2A–2D). We also observed qualitative changes in the Tfh compartment following IFNAR signaling blockade, including a 25% increase in Tfh expression of ICOS (Fig 2C and 2E), a co-stimulatory molecule that is essential for Tfh commitment, migration and function [37, 38]. These data support that *Plasmodium* infection-induced type I IFNs limit Tfh accumulation.

In addition to alterations in the Tfh compartment, α-IFNAR treatment also resulted in 20% decreases in both the amount of CD4 T cell-expressed T-bet and the proportion of T-bet^+^ Th1 effector CD4 T cells, compared to control MOPC treatment (Fig 2F and 2G). Notably, ectopic expression of T-bet can induce expression of Blimp-1 (*34*), a transcriptional repressor that regulates terminal differentiation of effector T cells (*35*) and limits Tfh and T cell effector function (*36*). Moreover, pathogen-specific T-bet^+^ effector CD4 T cells responding to prolonged or chronic infections often co-express Blimp-1 (*37*) and are functionally categorized as T regulatory 1 (Tr1) CD4 T cells. Thus, we further reasoned that type I IFNs also promote the expansion or accumulation of T-bet^+^Blimp-1^+^ Tr1 cells during experimental malaria. To formally test this, we infected Blimp-1-eYFP reporter mice with *P*. *yoelii* and examined the kinetics of simultaneous T-bet and Blimp-1-eYFP expression in *Plasmodium* infection-induced (S2A Fig) effector CD4 T cells. We found that ~50% of T-bet^+^ effector CD4 T cells co-expressed Blimp-1-eYFP in both MOPC-control and α-IFNAR-treated mice (Fig 2F). Notably, α-IFNAR treatment reduced the proportion and number of infection-induced effector CD4 T cells co-expressing both T-bet and Blimp-1-eYFP by >30% (Fig 2H–2J). Moreover, compared to effector CD4 T cells recovered from MOPC-treated mice, expression of Blimp-1-eYFP signal was reduced by >30% on days 8 and 16 p.i. in *Plasmodium* infection-induced effector CD4 T cells recovered from α-IFNAR-treated mice (Fig 2K).

To gain further insight into the link between type I IFN signaling and Blimp-1 induction, and determine whether the IFNα/β-Blimp-1 circuit is unique to *Plasmodium* infection, we next initiated comparative studies using mice infected with LCMV clone 13, which also triggers a highly inflammatory, Th1-biased response. On day 14 p.i., total virus infection-induced (CD44^hi^CD11a^hi^) and epitope-specific (GP_61-80_) effector CD4 T cells showed marked reductions in both the proportion of Blimp-1-eYFP^+^ cells (S2B Fig) and per-cell expression of Blimp-1-eYFP (S2C Fig) following administration of α-IFNAR to LCMV clone 13-infected mice. As a composite, these data show that type I IFNs suppress the development or accumulation of Tfh cells and promote the expansion of T-bet^+^Blimp-1^+^ effector CD4 T cells during blood stage *Plasmodium* infection and that the IFNα/β-Blimp-1 circuit is a generalizable feature of systemic, Th1-biased infections.

### Type I IFN signaling promotes the development and function of T regulatory 1 cells during experimental malaria

**Fig 3:**
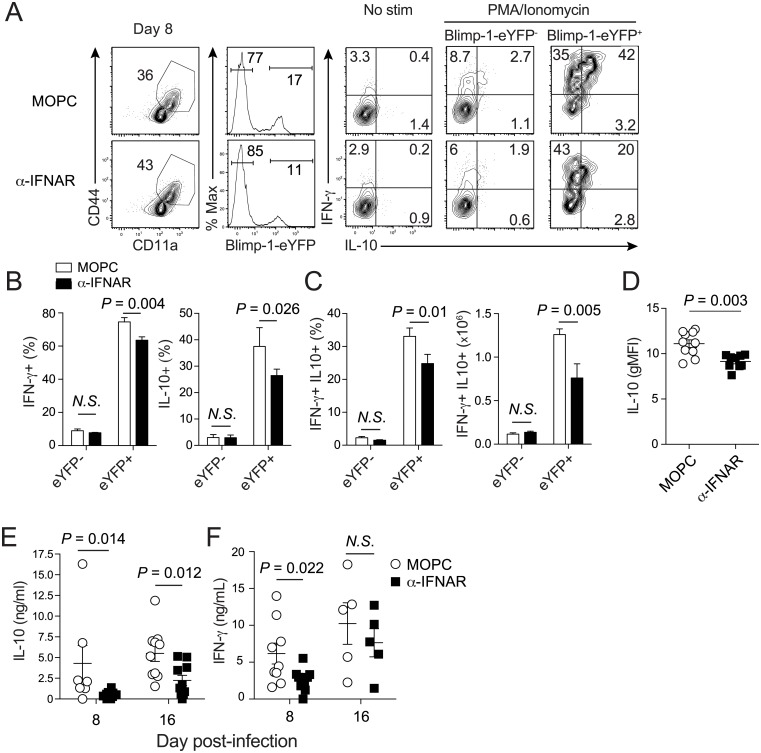
Type I interferons promote co-production of IFN-γ and IL-10 by *Plasmodium* infection-induced Tr1 cells. (**A-F**) Blimp-1-eYFP reporter mice were administered either MOPC or α-IFNAR antibodies and were infected with 10^6^ pRBCs. (**A**) Representative flow plots depicting the frequency of Blimp-1-eYFP expression and subsequent IFN-γ and IL-10 cytokine production in Blimp-1-eYFP^-^ and Blimp-1-eYFP^+^ splenic CD44^+^CD11a^hi^ CD4 T cells from MOPC and α-IFNAR-treated mice after ex vivo stimulation with PMA/Ionomycin. (**B**) Summary data displaying the proportion of Blimp-1-eYFP^-^ and Blimp-1-eYFP^+^ effector CD44^+^CD11a^hi^ CD4 T cells that are positive for either IFN-γ^+^ (left) or IL-10^+^ (right) after ex vivo stimulation. (**C**) Summary data displaying the proportion (left) and total number (right) of IFN-γ^+^ IL-10^+^ cells among Blimp-1-eYFP^-^ and Blimp-1-eYFP^+^ *Plasmodium* infection-induced CD4 T cells from MOPC and α-IFNAR-treated mice on day 8 p.i. (**D**) Summary data showing the gMFI of IL-10 in *Plasmodium* infection-induced CD4 T cells on day 8 p.i. (**E-F**) Summary data (Mean +/- SEM) showing the levels of circulating IL-10 (**E**) and IFN-γ (**F**) in sera from MOPC and α-IFNAR-treated mice. Data (Mean +/- SEM) in (**B-F**) are pooled from 2–3 independent experiments (3–4 mice/group per experiment) and were analyzed using Mann-Whitney non-parametric tests. Data in (**A-F**) are representative of 3 independent experiments. *N*.*S*. = not statistically significant.

To explore the biological consequences of reduced CD4 T cell co-expression of T-bet and Blimp-1 following α-IFNAR treatment we next examined cytokine production by *Plasmodium* infection-induced effector CD4^+^ Tr1 cells. Tr1 cells simultaneously express T-bet, Blimp-1, IFN-γ and IL-10 during chronic viral and protozoan infections and have been shown to sharply limit pathogen control [39–41]. Moreover, Foxp3-negative Tr1 CD4 T cells are the major source of IL-10 in these scenarios [39, 42], and Tr1 activity and expression of IL-10 is Blimp-1-dependent [42–44]. As predicted, co-expression of IFN-γ and IL-10 was largely restricted to Blimp-1-eYFP^+^ *Plasmodium* infection-induced effector CD4 T cells (Fig 3A), and α-IFNAR treatment significantly decreased the frequency and number of Blimp-1-eYFP^+^ IFN-γ^+^IL-10^+^ effector cells on day 8 p.i. (Fig 3A–3C) and the amount of IL-10 expressed per cell (Fig 3D). Consistent with alterations in T-bet^+^Blimp-1-eYFP^+^ Tr1 numbers (Fig 2J) and cytokine expression, we also observed 3-4-fold reductions in serum IL-10 levels and 2-fold reductions in serum IFN-γ between days 8–16 p.i. in α-IFNAR-treated *Plasmodium*-infected mice compared to MOPC-treated mice (Fig 3E and 3F). Of note, simultaneous expression of CD49b and LAG-3 is reported to identify Tr1 cells in both mice and humans [45]. Although the majority (>70%) of *Plasmodium* infection-induced Blimp-1-eYFP cells expressed LAG-3 (S3A and S3B Fig), CD49b expression was largely restricted to NK cells (S3C Fig). Collectively, these data show that early, *Plasmodium*-induced type I IFNs promote the development of effector Tr1 responses and Tr1 co-expression of IL-10 and IFN-γ during experimental malaria.

### IFN-γ and IL-10 collaborate to limit Tfh accumulation, humoral immunity and parasite control during *Plasmodium* blood-stage infection

**Fig 4:**
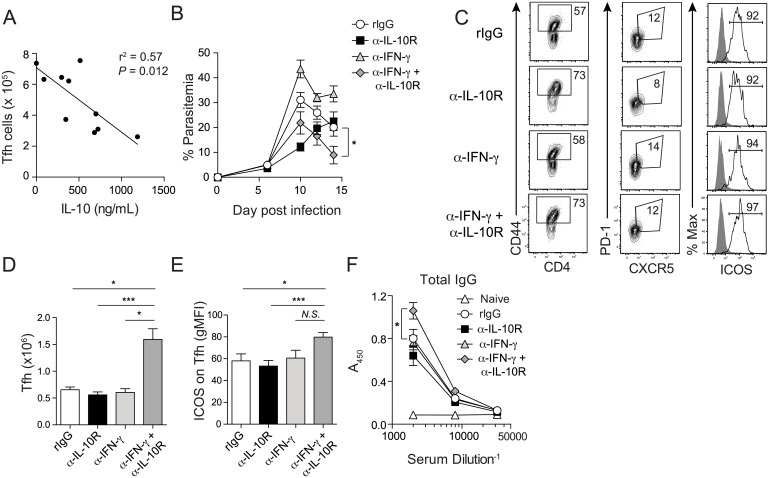
IFN-γ and IL-10 cooperate to limit Tfh accumulation and secreted antibody responses during *Plasmodium* blood-stage infection. (**A**) Negative correlation between levels of circulating IL-10 and the total number of splenic Tfh cells in MOPC and α-IFNAR-treated mice on day 16 p.i. Data were analyzed using linear regression. (**B-F**) Groups of mice (n = 5/group) were treated with either rIgG, α-IL-10R, α-IFN-γ, or α-IFN-γ+α-IL-10R. (**B**) Parasitemia kinetics. Statistical results for comparisons between rIgG- and α-IFN-γ+α-IL-10R-treated mice are displayed. Data were analyzed using Mann-Whitney non-parametric tests. (**C**) Representative flow plots depicting the proportion of PD-1^+^CXCR5^+^ Tfh cells among CD44^hi^ splenic CD4^+^ T cells in rIgG-, α-IL-10R-, α-IFN-γ or α-IFN-γ+α-IL-10R-treated mice. (**D**) Summary data showing the total number of splenic Tfh cells in rIgG-, α-IL-10R-, or α-IFN-γ+α-IL-10R-treated mice on day 14 p.i. (**E**) Summary data showing ICOS expression (gMFI) on Tfh cells from rIgG-, α-IL-10R-, α-IFN-γ or α-IFN-γ+α-IL-10R-treated mice. (**F**) Summary graph displaying the relative serum titers of MSP1_19_-specific total IgG in rIgG-, α-IL-10R-, α-IFN-γ, or α-IFN-γ+α-IL-10R-treated mice on d14 p.i. Statistical results for comparisons between rIgG- and α-IFN-γ+α-IL-10R-treated mice are displayed and data were analyzed using Mann-Whitney non-parametric tests. Summary data (Mean +/- SEM) in (**D-E**) were analyzed using Kruskal-Wallis non-parametric tests. Data in (**B-F**) are representative of two independent experiments (5 mice/group per experiment). *N*.*S*. = not statistically significant. *P<0.05, ***P<0.0001.

Excessive IFN-γ production by T cells limits Tfh and GC B cell activity and constrains anti-*Plasmodium* humoral immunity [15], supporting that IFN-γ can negatively regulate Tfh function. However, whether IL-10 can additionally or independently inhibit Tfh responses during *Plasmodium* infection has not been addressed. Indeed, the consistent improvements in parasite control and humoral immunity (Fig 1) and marked reductions in circulating serum and Tr1-expressed IL-10 and IFN-γ (Fig 3) following α-IFNAR treatment raised the possibility that together these cytokines may limit Tfh responses and humoral immunity during *Plasmodium* infection. In support of this notion, both serum IFN-γ [15] and IL-10 levels in experimental mice strongly and inversely correlated with the magnitude of the Tfh response on day 16 p.i (Fig 4A). Because α-IFNAR treatment significantly reduced the number of IL-10^+^IFN-γ^+^ *Plasmodium* infection-induced Tr1 cells and serum IFN-γ and IL-10 levels, we hypothesized that combinatorial IL-10R signaling blockade and IFN-γ neutralization would phenocopy α-IFNAR treatment, resulting in increased Tfh accumulation, improved humoral immunity, and enhanced parasite control during experimental malaria. We found that simultaneously blocking the activity of these Tr1-derived cytokines potently enhanced parasite control (Fig 4B), which was associated with expanded numbers of ICOS^+^ Tfh cells (Fig 4C–4E). Of note, GC B cell reactions were not significantly elevated by day 14 p.i. following simultaneous neutralization of IFN-γ and blockade of IL-10 signaling (S4A and S4B Fig), consistent with marginally altered GC B cell responses following α-IFNAR treatment at this early time point (Fig 1D) Nevertheless, combined targeting of both IFN-γ and IL-10 elevated titers of parasite-specific secreted antibody by day 14 p.i. (Fig 4F). Solely blocking IL-10 signaling transiently limited parasite replication (Fig 4B), but parasite control was eventually lost after day 14 p.i., which was associated with reduced GC B cell reactions (S4A and S4B Fig) and diminished secretion of parasite-specific antibody (Fig 4F). These data support that IL-10 insulates the host against the reported pathological effects of excess IFN-γ [15, 16, 43]. While neutralization of IFN-γ modestly enhanced GC B cell frequencies (S4B Fig), parasite control was also eventually impaired (Fig 4B). Indeed, improvements in both anti-*Plasmodium* humoral immunity and parasite control only occurred following simultaneous neutralization of IFN-γ and blockade of IL-10 signaling. As a composite, our data support that a primary effect of *Plasmodium* infection-induced type I IFN is the expansion of effector Tr1 cells and that Tr1-associated cytokines IL-10 and IFN-γ act together to limit Tfh accumulation, anti-malarial humoral immunity and parasite control via release of IL-10 and IFN-γ.

### Type I IFNs directly induce T-bet and Blimp-1 and co-expression of IL-10 and IFN-γ in *Plasmodium* infection-induced effector CD4 T cells during *Plasmodium* blood-stage infection

**Fig 5:**
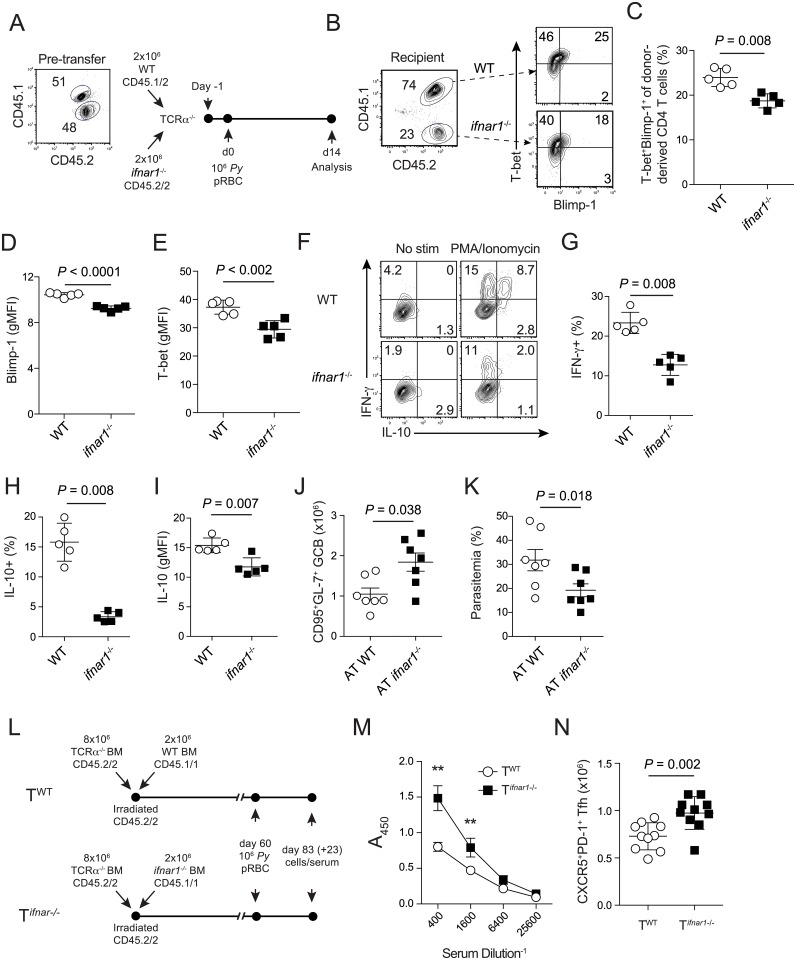
CD4 T cell intrinsic IFNAR signaling limits humoral immunity and parasite control via induction of Tr1 cell expression of T-bet and Blimp-1 and co-production of IFN-γ and IL-10. (**A**) Experimental design. *Tcrα* ^-/-^ mice were seeded with equivalent numbers of CD45.1/2 WT and CD45.2/2 (*ifnar1* ^-/-^) naïve CD4 T cells and infected with 10^6^ pRBCs one day post-transfer. Cellular reactions were analyzed 2 weeks p.i. (**B**) Representative flow plots showing the proportion of WT and *ifnar1* ^-/-^ cells among recovered activated CD44^hi^ CD4^+^ T cells and their simultaneous expression of T-bet by Blimp-1 on day 14 p.i. (**C**) Summary graph depicting the proportion of activated WT and *ifnar1* ^-/-^ CD4 T cells simultaneously expressing both T-bet and Blimp-1. (**D-E**) Summary graphs displaying the relative expression (gMFI) of Blimp-1 (**D**) and T-bet (**E**) in activated WT and *ifnar1* ^-/-^ CD4 T cells. (**F**) Representative flow plots depicting the proportion of activated WT and *ifnar1* ^-/-^ CD4 T cells competent to produce IFN-γ and IL-10 after ex vivo stimulation. (**G-I**) Summary data displaying the frequency of activated WT and *ifnar1* ^-/-^ CD4 T cells producing either IFN-γ (**G**) or IL-10 (**H**) on day 14 p.i. after ex vivo stimulation. (**J-K**) Separate groups of *tcrα* ^-/-^ mice were seeded with equivalent numbers of WT (CD45.1/2) and *ifnar1* ^-/-^ (CD45.2/2) naïve CD4 T cells and infected with 10^6^ pRBCs once day post-transfer. Parasite burdens and cellular reactions were analyzed on day 16 p.i. (**L**) Experimental design for generating mixed bone marrow chimeras in which the T cell compartment is either WT (T^WT^) or *ifnar*-deficient (T^*ifnar-/-*^). (**M**) Secreted parasite-specific IgG was evaluated by ELISA in chimeric mice on day 23 p.i.. (**N**) Summary data depicting the total number of Tfh cells in chimeric mice. Data (Mean +/- SD) in (**C-E, G-I, and M,N**) were analyzed using Mann-Whitney non-parametric tests and are representative of two independent experiments with 5 mice per group per experiment. Data (Mean +/- SEM) in (**J,K**) were pooled from two independent experiments with 3–4 mice/group per experiment and were analyzed using Mann-Whitney non-parametric tests.

Our data show that neutralizing either type I IFN or Tr1 effector cytokines IFN-γ and IL-10 promotes the expansion of Tfh cells and enhances humoral immunity and parasite control. However, to formally test whether type I IFN-mediated suppression of Tfh accumulation and/or induction of the T-bet-Blimp-1 axis in responding CD4 T cells is CD4 T cell-intrinsic, we seeded naive *tcrα* ^-/-^ mice with equivalent numbers of naïve, congenically marked wild type (WT) and *ifnar1* ^-/-^ CD4 T cells one day before *P*. *yoelii* challenge (Fig 5A). At defined intervals after challenge, we assessed Tfh Bcl-6 expression, as well as T-bet and Blimp-1 co-expression in effector WT and *ifnar1* ^-/-^ CD4 T cells recovered from the same host. Although *ifnar1* ^-/-^ CD4 T cells did not expand or accumulate to the same degree as WT CD4 T cells (S5A and S5B Fig) we found no difference in the proportion of WT and *ifnar1* ^-/-^ donor-derived cells that adopted the canonical CXCR5^+^PD-1^hi^Bcl-6^+^ Tfh phenotype (S5A–S5D Fig), suggesting that direct IFNAR signaling on CD4 T cells does not intrinsically impair Tfh differentiation during experimental malaria. In line with this, expression of IFNAR was negligible on effector Tfh cells, compared to effector Th1/Tr1 effector CD4 T cells (not depicted). Decoupling of IFNAR signaling in expanding Tfh cells would also be consistent with reports showing that Tfh cells become refractory to specific cytokine signaling networks very early after their initial priming [46]. By contrast, we found markedly lower proportions of T-bet^+^Blimp-1^+^ *ifnar1* ^-/-^ CD4 T cells (Fig 5B and 5C), as well as 20–40% less T-bet and Blimp-1 in *ifnar1* ^-/-^ CD4 T cells, compared to WT CD4 T cells (Fig 5D and 5E). Notably, the proportionate decreases in T-bet and Blimp-1 expression in *ifnar* ^-/-^ CD4 T cells mirror those observed following anti-IFNAR-treatment (Fig 2H and 2I) and co-adoptive transfer studies also revealed sharp reductions in IFN-γ, and IL-10 expression by *ifnar1* ^-/-^ effector CD4 T cells, compared to WT CD4 T cells recovered from the same host (Fig 5F–5I), confirming that the impact of IFNα/β signaling on IL-10 and IFN-γ expression are CD4 T cell-intrinsic. These data support that type I IFN-mediated induction of the Tr1 program during experimental malaria is primarily regulated by CD4 T cell-intrinsic IFNAR signaling. To determine the impact of CD4 T cell-intrinsic *ifnar1*-deficiency on anti-*Plasmodium* humoral immunity, we undertook two complementary chimeric approaches. First, transfer of WT or *ifnar1* ^-/-^ naïve CD4 T cells into separate groups of *tcrα* ^-/-^ mice (e.g. Fig 5A) revealed that both *Plasmodium* infection-induced GC B cell responses and parasite control were significantly enhanced in mice seeded with *ifnar1* ^-/-^ CD4 T cells (Fig 5J and 5K). Second, and consistent with our chimeric transfer studies, infection of mixed bone marrow chimeras in which *ifnar1* deficiency was restricted to the T cell compartment (Fig 5L) revealed 2-fold greater parasite-specific secreted IgG (Fig 5M), as well as elevated Tfh (Fig 5N and S5E Fig), and GC B cell responses (S5F and S5G Fig). Collectively, these data support that type I IFNs indirectly limit the accumulation of *Plasmodium*-specific Tfh cells and impede humoral immunity and parasite control during experimental malaria via the activity of Tr1 effector cytokines. Mechanistically, although *Plasmodium* infection-induced type I IFNs have no appreciable direct effects on modulating Bcl-6^+^ Tfh development during malaria, they directly promote the accumulation and function of *Plasmodium* infection-induced T-bet^+^Blimp-1^+^ Tr1 cells that limit humoral immunity and parasite control via secretion of effector cytokines IL-10 and IFN-γ.

### Type I IFN induction of Blimp-1 can occur independently of IL-2 signaling

**Fig 6:**
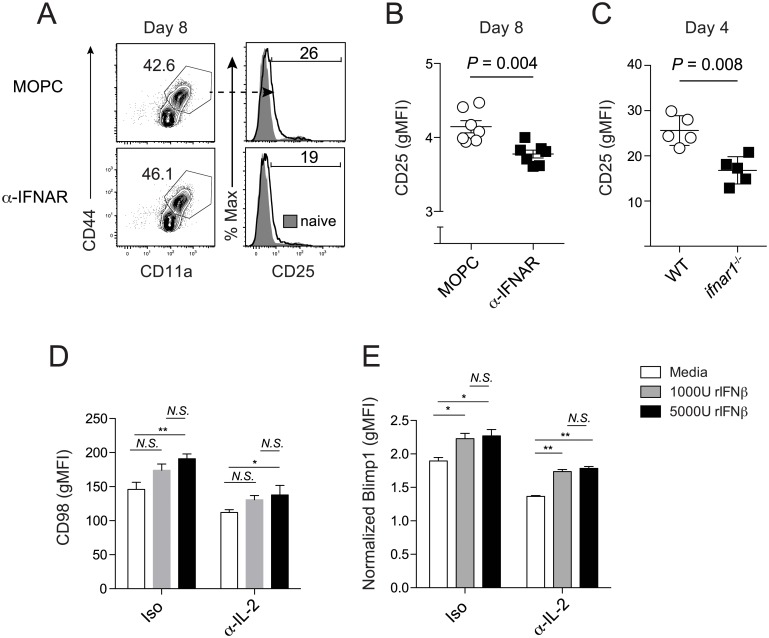
Type I IFN-mediated induction of Blimp-1 in CD4 T cells can occur independently of IL-2 signaling. (**A-B**) Blimp-1 reporter mice were administered either MOPC or α-IFNAR antibodies and were infected with either10^6^ pRBCs. (**A**) Representative flow plots depicting the expression of CD25 on *Plasmodium* infection-induced effector CD4 T cells from MOPC and α-IFNAR-treated mice on day 8 p.i. (**B**) Cumulative data showing surface CD25 expression (gMFI) on *Plasmodium* infection-induced splenic CD44^hi^CD11a^hi^ CD4^+^ T cells. Data (Mean +/- SEM) in (**B**) are pooled from 2 independent experiments (3–4 mice/group). (**C**) Summary data (Mean +/- SD) showing CD25 expression (gMFI) on WT and *ifnar1* ^-/-^ CD4 T cells recovered from *tcrα* ^-/-^ on day 4 post-*Plasmodium* infection. Data in (B,C) were analyzed using Mann-Whitney non-parametric tests of statistical significance. (**D,E**) Naïve CD4 T cells from Blimp-1-eYFP reporter mice were stimulated with α-CD3/α-CD28 and treated with or without rIFNβ or anti-IL-2 blocking antibodies. (**D**) Summary data (Mean +/- SD) depicting surface expression of PI3K-dependent CD98 on CD44^hi^ CD4^+^ T cells cultured under the various treatment conditions. (**E**) Summary data (Mean +/- SEM) showing Blimp-1-eYFP expression (gMFI) in CD44^hi^ CD4^+^ T cells under the various treatment conditions. Data in (**E**) are normalized to Blimp-1 expression (MFI) in non-stimulated CD4 T cells and are pooled from 3 independent experiments. Data in (D,E) are representative of 3 independent experiments and were analyzed using Kruskal-Wallis non-parametric tests of statistical significance. *N*.*S*. = not statistically significant. *P<0.05, **P<0.01.

CD4 T cell-intrinsic IFNAR signaling can induce expression of the high-affinity IL-2 receptor (CD25), sensitizing CD4 T cells to IL-2 signaling [47] and activation of STAT5 [23] a known inducer of *prdm1*, which encodes for Blimp-1. To begin to explore the mechanistic role of IL-2 in the IFNα/β-Blimp-1 circuit, we examined the expression kinetics of CD25 on *Plasmodium* infection-induced and LCMV GP_61-80_-specific CD4 T cells in MOPC- and α-IFNAR-treated *Plasmodium* parasite- or virus-infected mice, respectively. We found that α-IFNAR treatment significantly reduced CD25 expression on CD4 T cells responding to either *Plasmodium* (Fig 6A and 6B) or LCMV clone 13 infection (S6A and S6B Fig). Notably, reduced Blimp-1 expression in co-transferred *ifnar1* ^-/-^ CD4 T cells (Fig 5B–5D) also correlated with a 30% decrease in CD25 surface expression compared to WT effector CD4 T cells on day 4 p.i. (Fig 6C), suggesting that type I IFNs may function to elevate or sustain CD25 expression, thereby enhancing STAT5 activity and Blimp-1 induction. To formally test this, we next performed *in vitro* CD4 T cell culture experiments. We observed dose-dependent effects of exogenous IFNβ-mediated phosphatidylinositol 3-kinase (PI3K) activation and Blimp-1 induction, as measured by CD98 expression [48] and Blimp-1-eYFP expression, respectively, in CD4 T cells (Fig 6D and 6E). Strikingly, IFNβ-mediated induction of Blimp-1 and PI3K activation occurred even when IL-2 was neutralized (Fig 6D and 6E). These results support that type I IFNs may directly induce Blimp-1 expression in CD4 T cells independently of IL-2 signaling, likely via activation of the PI3K pathway. Collectively, our results show that CD4 T cell intrinsic type I IFN signaling directly induces T-bet and Blimp-1 expression in *Plasmodium* infection-induced effector CD4 T cells, thereby promoting Tr1 cell development and function. Moreover, Tr1 effector cytokines IL-10 and IFN-γ collaborate to suppress Tfh accumulation, anti-*Plasmodium* humoral immunity, and parasite control.

## Discussion

The importance of IL-10-secreting Tr1 cells in limiting immunopathology during highly inflammatory Th1-biased infection is well appreciated [40, 41, 43, 49]. However, the pathways that induce or support Tr1 differentiation are not well understood. Here we show that early type I IFN responses during inflammatory Th1-biased infections promote the differentiation of T-bet^+^Blimp-1^+^ Tr1 cells and limit humoral immunity and parasite control during experimental malaria. Mechanistically, we identified that type I IFNs directly induce T-bet and Blimp-1 expression in CD4 T cells responding to *Plasmodium* blood-stage infection, which amplifies their production of IL-10 and IFN-γ. Furthermore, we show that these two Tr1-associated cytokines collaborate to suppress humoral immunity and parasite control.

Multiple reports show that type I IFNs are highly induced during malaria, yet the extent to which they regulate anti-*Plasmodium* immunity or disease is often conflicting. Clinical studies show that specific SNPs in the gene encoding *ifnar1* are associated with resistance to cerebral malaria [50], and case-control studies in Angolian children and neuro-malaria patients in Thailand support that type I IFN responses are either associated with the development of cerebral malaria [51] or precede the induction of IFN-γ expression and severe disease [20]. These latter studies are consistent with our experiments showing that blocking type I IFN signaling during non-lethal experimental malaria abrogates the development of IFN-γ^+^T-bet^+^ Th1 responses. Similarly, multiple reports show that *ifnar1* ^-/-^ mice have altered Th1 activity and are largely resistant to the development of acutely lethal *P*. *berghei*-induced experimental cerebral malaria (ECM) [17, 21, 25, 52]. In contrast, following infection with non-lethal *P*. *yoelii* or *P*. *chabaudi* spp., *ifnar1* ^-/-^ mice exhibit either exacerbated disease and higher parasite burdens [22, 53] or no phenotype at all [18]. Although most studies have focused on the role of type I IFN signaling in either regulating myeloid dendritic cell stimulatory potential or *Plasmodium* parasite control during the first week of acute infection, to our knowledge no studies have examined whether type I IFN responses regulate humoral immunity during *Plasmodium* infection. To address this question, and to avoid pitfalls associated with altered immune system development or dysregulated hematopoiesis in *ifnar1* ^-/-^ mice [54, 55], we employed transient blockade of type I IFN responses and chimeric/genetic approaches to evaluate CD4 T cell intrinsic and extrinsic roles for type I IFN signaling during experimental malaria. We found that IFNAR signaling blockade during experimental malaria enhanced the quantity and quality of the Tfh cell response, bolstered the magnitude of the germinal center reaction and elevated titers of parasite-specific antibody against MSP1_19_, all of which were associated with improved control over parasite replication. Our chimeric studies show that type I IFN-mediated suppression of Tfh accumulation and humoral immunity is indirect.

During chronic LCMV infection, blocking type I IFN signaling, primarily the activity of IFNβ [56], enhances CD4 T cell function and accelerates resolution of persistent infection [27, 28]. However, the two arms of the adaptive immune response primarily responsible for limiting virus persistence and promoting resolution of LCMV, cytotoxic CD8 T cells and Tfh-dependent antibody responses, were not quantitatively or qualitatively enhanced [27, 28]. By comparison, during acute LCMV infection, type I IFNs are purported to directly inhibit Tfh cell differentiation via up-regulation of CD25, which sensitizes CD4 T cells to IL-2 signaling and promotes STAT5 binding to the Bcl-6 promoter, thereby blocking the Tfh-promoting activity of STAT3 [23]. Of note, administration of recombinant IFNβ during *in vitro* activation of naïve CD4 T cells induced CXCR5 and Bcl-6 expression in WT but not *ifnar1* ^-/-^ CD4 T cells (not depicted and [24], highlighting that type I IFNs may positively regulate molecules important for the Tfh cell program in vitro. Thus, we examined possible CD4 T cell-intrinsic impacts of type I IFN signaling during experimental malaria. To do this, we performed co-adoptive transfer studies of WT and *ifnar1* ^-/-^ CD4 T cells into *Plasmodium* infected mice, because analyses of humoral immunity and parasite control in *tcrα* ^-/-^ mice seeded with either WT or *ifnar1* ^-/-^ cells may be confounded by the markedly reduced proliferation of CD4 T cells lacking the type I IFN receptor (e.g. Fig 5C). We found *ifnar1* ^-/-^ and WT CD4 T cells differentiated into Tfh cells in equal proportions, despite reduced CD25 expression on *ifnar1* ^-/-^ CD4 T cells. These data suggest that neither CD4 T cell-intrinsic type I IFN signaling nor IFN-mediated upregulation of CD25 directly limits Tfh development during experimental malaria. Instead, our data show that *Plasmodium* infection-induced type I IFNs promote the differentiation and function of T-bet^+^Blimp-1^+^ effector CD4 T cells. Pathogen-specific effector CD4 T cells are the major source of IL-10 following prolonged viral and parasitic infection. We found that IL-10 and IFN-γ expression were largely restricted to Blimp-1^+^ Tr1 cells, compared to all other *Plasmodium* infection-induced effector CD4 T cell subsets. Moreover, we identified that the effector cytokines of Tr1 cells, IL-10 and IFN-γ, constrain Tfh accumulation and protective humoral immunity. Our data supporting IL-2-independent induction of Blimp-1 during *Plasmodium* infection is also consistent with a recent report showing that IL-2 blockade failed to modulate Blimp-1 expression in LCMV-specific CD4 T cells [42].

Excessive Th1 activity and secretion of IFN-γ can limit the development and function of Tfh cells during malaria [15, 16, 57]. As noted, type I IFNs can increase CD4 T cell responsiveness to IL-2 signaling, which can stimulate STAT5 activation and skew CD4 T cells towards Th1 lineage commitment. Alternatively, *in vitro* studies show that type I IFNs can activate STAT4-mediated induction of T-bet and Th1 activity [24]. Consistent with these observations, we found that blocking type I IFN signaling during experimental malaria reduced T-bet expression in infection-induced Th1 cells. Here we extend these data and provide *in vivo* evidence that type I IFNs may directly induce T-bet to reinforce the Th1 program. Notably, ectopic expression of T-bet in antigen-specific CD8 T cells promotes Blimp-1 expression [58]. Furthermore, several studies show that during prolonged, highly inflammatory parasitic and viral infections, Th1 cells co-express the transcription factor Blimp-1, a transcriptional repressor that is required for Th1/Tr-1 production of IL-10 [42–44]. Tr1 cell production of IL-10 is known to limit pathogen control during chronic parasitic and viral infections [42, 43]. Thus, we assessed whether type I IFNs also impact Blimp-1 expression in CD4 T cells during experimental malaria or chronic LCMV infection. Strikingly, IFNAR signaling blockade decreased Blimp-1 expression in both parasite infection induced- and virus-specific CD4 T cells. Thus, the type I IFN-Blimp-1 axis represents a generalizable feature of prolonged/chronic pro-inflammatory infections. Importantly, compared to WT CD4 T cells, *ifnar1* ^-/-^ CD4 T cells responding to malaria also exhibited a 30% decrease in the co-expression of T-bet and Blimp-1, demonstrating that CD4 T cell-intrinsic IFNAR signaling directly induces transcription factors important for Tr1 function. Accordingly, both antibody-mediated blockade and genetic ablation of IFNAR signaling resulted in markedly reduced Tr1 expression of IFN-γ and IL-10. These effects were also accompanied by reduced levels of serum IFN-γ and IL-10 in α-IFNAR-treated mice.

The importance of Tr1 cells in limiting inflammatory-mediated immunopathology during chronic viral and parasitic infections has been established [39, 40, 43, 59, 60]. However, Tr1 cell production of IL-10 can also hamper pathogen control [42, 43]. Defining the cellular and molecular mechanisms that govern Tr1 cell differentiation and function is therefore of interest and has therapeutic implications for Th1-inflammatory and autoimmune diseases, including cancer. Previous reports have identified that the inflammatory cytokines IL-27 and IL-12 drive Blimp-1 and IL-10 expression in both antigen-specific Tr1 and CD8 T cells. [61–63]. Furthermore, ICOS is linked to Tr1 development and activity [64], and recent studies show that systemic IL-27 and ICOS cooperate to regulate Tr1 activity during experimental malaria [65]; however, in these reports neither IL-27R signaling nor ICOS was essential for Tr1 development during *P*. *yoelii* malaria [65]. By contrast, we show that type I IFNs act directly on pathogen-specific CD4 T cells to induce co-expression of T-bet and Blimp-1 and their downstream Tr1-assocatied effector molecules IFN-γ and IL-10. Our in vivo data are also consistent with studies demonstrating IFNβ can trigger IL-10 production by CD4 T cells in vitro [66]. While CD4 T cell-derived IL-2 has been linked to Blimp-1 and IL-10 expression in CD8 T cells [67], we found that IFNβ-mediated Blimp-1 induction in *in vitro* stimulated CD4 T cells did not involve IL-2 signaling. Thus, our data uncover a previously unrecognized circuit and an additional mechanism for Tr1 development in vivo during highly inflammatory, Th1-biased infections.

One of the most striking and consistent differences between *Plasmodium* infection-induced effector CD4 T cells recovered from control rIgG- and α-IFNAR-treated mice, or WT and *ifnar1* ^-/-^ effector CD4 T cells following adoptive transfer, was their diminished capacity to co-produce IL-10 and IFN-γ when IFNAR signaling was abrogated. While excessive IFN-γ is known to limit anti-*Plasmodium* humoral immunity [15, 57], only a limited number of studies have examined the impact of IL-10 on the germinal center reaction and humoral immunity, with variable results reported [68–72]. Although IL-10 can constrain Tfh differentiation following immunization [69], whether IL-10 plays a role in promoting or inhibiting Tfh cell differentiation, maintenance or function during infection remains less clear. Notably, a recent study identified that frequencies of IFN-γ and IL-10 co-producing CD4 T cells were increased in Ugandan children who presented with >2 malaria episodes/year, compared to children who presented with <2 malaria episodes/year [73]. Although this study did not assess humoral immunity (nor could causality be determined), these observations are consistent with Tr1 responses limiting protective immunity.

The capacity of IL-10 to modulate humoral immunity is likely multifaceted. Our data show that blockade of IL-10R signaling abrogates humoral immunity during experimental malaria. However, simultaneously blocking IL-10R signaling and neutralizing IFN-γ enhances parasite control and parasite-specific antibody responses. IL-10 can also limit the activity and function of antigen presenting cells (APC) [74], with potential impacts on Tfh priming. Conversely, IL-10 can signal via STAT3, which is important for promoting Tfh development [23]. Of note, IL-10 can also function in a feedback loop to limit the activity of effector Th1 cells during chronic virus infection [42]. In that study, IL-10 expression was Blimp-1-dependent, and prolonged TCR engagement and IRF4 activity were mechanistically linked to Blimp-1 induction. Our data show that type I IFN signaling directly and additionally contributes to Blimp-1 induction in cells responding to either *Plasmodium* or chronic LCMV infection, further supporting that type IFN-mediated induction of Blimp-1 and subsequent expansion of immunosuppressive Tr1 cells represents a generalizable feature of inflammatory Th1-biased infections.

Collectively, our results identify a previously unrecognized inhibitory circuit wherein CD4 T cell intrinsic type I IFN-signaling directly induces a T-bet-Blimp-1 axis in responding *Plasmodium* infection-induced Tr1 cells, thereby promoting expression of IFN-γ and IL-10 that cooperate to inhibit Tfh accumulation and expression of ICOS. Our data also support that blockade of IFNAR signaling or neutralization of Tr1 effector molecules IFN-γ and IL-10 during malaria may bolster *Plasmodium*-specific Tfh responses, enhance parasite control, and promote the establishment of humoral immunity. Our report identifies additional mechanisms that limit *Plasmodium*-specific antibody responses and highlights pathways that warrant consideration as potential targets when designing an efficacious malaria vaccine or novel immunotherapeutics to combat malarial disease.

## Materials and Methods

### Ethic statement

All animal experiments were conducted in accordance with the Animal Welfare Act and the recommendations in the Guide for the Care and Use of Laboratory Animals of the National Institutes of Health. OUHSC animal facilities have full accreditation from the Association for Assessment and Accreditation of Laboratory Animal Care and are PHS-assured (Assurance Number: # A3165-01). All animal procedures were approved by the OUHSC Animal Care and Use Committee (IACUC) under protocol 15–088 and the Office of Animal Welfare Assurance (OAWA), which oversees the administration of the IACUC at OUHSC.

### Mice, *Plasmodium* and LCMV infection, and biologics

C57BL/6 wild type, *tcrα* ^-/-^, Blimp-1-eYFP reporter, *ifnar1* ^-/-^, and *il10rb* ^-/-^ mice (6–8 weeks, 16–21 g) were purchased from Jackson Laboratories. *Plasmodium yoelii* (clone 17XNL, obtained from MR4 (ATCC)) was routinely passaged through mosquitoes and mouse infections were initiated by serial transfer of 10^6^ parasite-infected red blood cells via tail vein injection. LCMV clone 13 infection was administered intravenously at a dose of 10^6^ PFU. Parasitemia was measured using flow cytometry as described [75]. One day prior to *P*. *yoelii* infection, mice were injected i.p. with either 1.5 mg of MOPC isotype control or MAR1-5A3 (α-IFNAR) monoclonal antibodies, followed by 0.75 mg doses on days 2 and 4 p.i. Anti-IL-10 receptor (clone 1B1.3a), anti-IFN-γ (clone XMG1.2) were injected i.p. in 200 and 500 μg doses on days 7 and 10 p.i., respectively. All biologics were acquired from BioXcell.

### Bone marrow chimeras

For T^WT^ and T^*ifnar-/-*^ chimeras, WT recipient mice were irradiated with 6.5 and 5.5 Gy, separated by 12 hours. Bone marrow from *tcrα* ^-/-^ and *ifnar* ^-/-^ or WT mice was mixed 1:9 and 10^7^ cells were injected i.v. Mice were maintained on oral sulfamethoxazole for 2 weeks. Chimerism was assessed at 6 weeks in peripheral blood using congenic markers. Chimerism in the T cell compartment was 65–70% among 20 mice in two independent experiments. Mice were infected with *P*. *yoelii* at 8 weeks.

### Flow cytometry

Mouse splenocytes were subjected to red blood cell lysis, washed and subsequently stained using fluorescently labeled antibodies against mouse CD4 (clone GK1.5), CD11a (clone M17/4), CD19 (clone 6D5), CD25 (clone PC61), B220 (clone RA3-6B2), CD44 (clone IM7), CD49b (clone DX5), CD95 (clone Jo2), CD98 (clone RL388), CD138 (clone 281–2), ICOS (clone 7E.17G9), LAG-3 (clone eBioC9B7W), PD-1 (clone RMP1-30), T and B cell activation antigen (clone GL-7), IgD (clone 11-26c.2a), or IgM (clone RMM-1). Reagents were acquired from Biolegend, Tonbo, eBioscience or BD Bioscience. Mouse splenocytes from LCMV cl13-infected mice were also stained with GP_61-80_ tetramer reagents (NIH) for one hour at room temperature before performing additional surface staining. In some experiments, cells were permeabilized with cytofix/cytoperm (BD Bioscience) followed by intracellular staining using anti-mouse IL-10 (JES5-16E3) and anti-mouse IFN-γ, (XMG1.2; Biolegend) or anti-IL-2 (JES6-5H4; eBioscience). For analysis of T follicular helper cells, splenocytes were incubated for 60 min at 4°C with rat anti-mouse CXCR5 (2G8; BD) and subsequently stained for 30 min at 4°C in the presence of biotinylated goat anti-rat IgG (Jackson), followed by staining with fluorochrome-conjugated anti-CD4, anti-CD44, anti-PD-1, and streptavidin-APC. T-bet (4B10), Bcl-6 (K112-91), and Blimp-1 (5E7), were stained after fixation and permeabilization using the FoxP3 staining buffer set (eBioscience). Samples were acquired using a Stratedigm S1200Ex flow cytometer and data was analyzed using FlowJo software (Tree Star, Inc., Ashland OR).

### MSP1_19_ and Cytokine ELISA

Plates (Nunc) were coated with recombinant MSP1_19_ (MR4) blocked with 2.5% BSA/5% normal goat serum and MSP119-specific IgG was detected in pre-diluted serum samples using HRP-conjugated goat anti-mouse-IgG, -IgG2b, or -IgM (Jackson ImmunoResearch). The SureBlue Reserve TMB Kit (KPL) was used as substrate and absorbance was analyzed with a Spectra Max 340 (Molecular Devices). For serum cytokine analyses, plates were coated with 2 μg/mL of IL-10 or IFN-γ capture antibodies (eBioscience), blocked with 2.5% BSA/5% fetal calf serum. Serum samples were subsequently applied at a 1:4 dilution. Wells were washed, incubated with biotinylated detection antibodies (eBioscience) and developed with streptavidin-HRP at room temperature for 30 minutes before applying SureBlue Reserve TMB substrate as described above.

### In vitro T cell culture

Naïve CD4 T cells were enriched from spleens of Blimp-1-eYFP reporter mice and stimulated with plate-bound α-CD3/α-CD28 for 3 days while treating with or without 1000 U/ml or 5000 U/ml of rIFNβ +/- blocking antibodies against IL-2 (JES6-1A12; used at a concentration of 10 μg /mL).

### Statistical analyses

Statistical analyses were performed using GraphPad Prism 6 software (GrapPad). Specific tests of statistical significance are detailed in figure legends.

## Supporting Information

S1 Fig **(A) Mice were infected with 10** ^**6**^ ***P*. *yoelii* infected red blood cells (pRBCs) and then administered either MOPC (isotype) or α-IFNAR antibodies at the indicated time points (arrows).** Parasitemia (% of RBCs infected) kinetics from two independent experiments are shown. (**B-C**) Representative dot plots illustrating that GC B cell (B) and Tfh (C) responses are *Plasmodium* infection-induced.
(EPS)

S2 Fig **(A) Representative flow plots depicting the proportion of CD44** ^**hi**^ **Blimp-1-eYFP** ^**+**^ **splenic CD4 T cells in naïve and *Plasmodium* infected mice.** (**B**) Representative flow plots depicting the proportion of splenic GP_61-80_ specific CD4 T cells (left) and their subsequent expression of Blimp-1-eYFP (right) 14 days following LCMV infection. (**C**) Summary data (n = 5 mice/group) showing Blimp-1-eYFP expression (gMFI) in GP_61-80_ specific and effector (CD44^hi^CD11a^hi^) CD4 T cells on day 14 post LCMV cl13 infection.
(EPS)

S3 Fig **(A-B). Analyses of LAG-3 and CD49b expression on *Plasmodium* infection-induced CD44** ^**hi**^ **Blimp-1-eYFP** ^**+**^ **splenic CD4 T cells on day 10 p.i. (C) Positive control staining to verify CD49b reagent, which is also a pan-NK cell marker.** NK cells were identified via NK1.1 and CD3ε staining.
(EPS)

S4 Fig **(A) Representative flow plots showing the frequency of splenic GC B cells in rIgG-,** α**-IL-10R-,** α**-IFN-γ, or** α**-IFN-γ+**α**-IL-10R-treated mice on d14 p.i.** (**B**) Summary data (n = 5 mice/group) depicting the frequency of GC B cells on day 14 p.i.
(EPS)

S5 Fig **(A) Representative dot plots and histograms depicting Tfh differentiation among WT and *ifnar*** ^**-/-**^ **CD4 T cells recovered from the same *Plasmodium*-infected *tcrα*** ^**-/-**^ **mice on day 14 p.i.** (**B**) Summary graph displaying the total number of recovered WT and *ifnar1* ^-/-^ CD44^hi^ effector CD4^+^ T cells. (**C-D**) Summary data showing proportion of Tfh cells (**C**) among recovered WT and *ifnar1* ^-/-^ CD44^hi^ CD4^+^ T cells and their expression of Bcl-6 (gMFI) (**D**). (**E-F**) Representative dot plots depicting the proportion of PD-1^hi^CXCR5^+^ Tfh cells (E) and GC B cells (F) recovered on day 23 p.i. from *Plasmodium* infected mixed bone marrow chimeric mice with either WT (T^WT^) or *ifnar* ^-/-^ (T^*ifnar*-/-^) T cell compartments. (**G**) Summary data depicting the total number of GC B cells in *Plasmodium* infected T^WT^ and T^*ifnar*-/-^ mice.
(EPS)

S6 Fig **(A) Representative flow plots depicting the proportion of GP**_**61-80**_ **specific CD4 T cells and their subsequent expression of CD25 (right) on day 14 post LCMV clone 13 infection.** (**B**) Cumulative data (Mean +/- SD) showing the relative expression of CD25 (gMFI) on GP_61-80_ specific and splenic effector (CD44^hi^CD11a^hi^) CD4^+^ T cells on day 14 post LCMV clone 13 infection from control and α-IFNAR-treated mice.
(EPS)
